# Assessment of Genetic Variation in *Apis nigrocincta* (Hymenoptera: Apidae) in Sulawesi Revealed by Partial Mitochondrial Cytochrome Oxidase I Gene Sequences

**DOI:** 10.1155/2020/1609473

**Published:** 2020-04-07

**Authors:** Christian A. Lombogia, Jimmy Posangi, Hard N. Pollo, Max Tulung, Trina E. Tallei

**Affiliations:** ^1^Entomology Study Program, Postgraduate Program, Universitas Sam Ratulangi, Manado, North Sulawesi, Indonesia; ^2^Nursing Study Program, Faculty of Nursing, Universitas Katolik De La Salle, Manado, North Sulawesi, Indonesia; ^3^Public Health Study Program, Faculty of Public Health, Universitas Sam Ratulangi, Manado, North Sulawesi, Indonesia; ^4^Forestry Science Study Program, Faculty of Agriculture, Universitas Sam Ratulangi, Manado, North Sulawesi, Indonesia; ^5^Department of Biology, Faculty of Mathematics and Natural Sciences, Universitas Sam Ratulangi, Manado, North Sulawesi, Indonesia

## Abstract

Asian cavity-nesting honey bee *Apis nigrocincta*, a native bee species from Sulawesi and the Philippines, plays a vital role in pollinating flowering plants in local ecosystem and agriculture. In this study, we assessed the intraspecific genetic variation of *A. nigrocincta* using the sequence of cytochrome c oxidase subunit I (COI). Molecular phylogenetic analysis showed that there were three main clades in *A. nigrocincta* specimens from Sulawesi based on their respective locations (North, Central, and South Sulawesi). Genetic distance analysis using the Kimura 2-parameter (K2P) model showed that the intraspecific genetic distance in Sulawesi specimens ranged from 0.000 to 0.055. There are 26 nucleotide polymorphic sites within Sulawesi *A. nigrocincta*. The variation was dominated by transition T ↔ C. The molecular identification result was supported by morphological identification. The results of the two methods agree that the specimen under study was *A. nigrocincta*. The result of genetic distance calculation indicated that although the tested specimens were derived from remote locations, the genetic variation was still within the range of intraspecific variation.

## 1. Introduction

There are about 30,000 bee species distributed worldwide. About 17,000 of them have been described [1]. Of them, about 20,000 species belong to the superfamily Apoidea [2]. Apidae, the largest family within this superfamily, contains at least 5,700 species of bees [3]. Honey bees are included in the genus *Apis*. These insects play a significant role in the pollination of important crops. Currently, there are nine species of honey bees known to inhabit the world: *Apis dorsata*, *A. laboriosa*, *A. mellifera*, *A. florea*, *A. andreniformis*, *A. cerana*, *A. koschevnikovi*, *A. nigrocincta* (Sulawesi, Indonesia, and Mindanao, the Philippines), and *A. nuluensis* (Kalimantan, Indonesia), with approximately 44 subspecies [4].

The honey bees are prone to extinction due to their haplodiploid sex-determining mechanism, limited population because of anthropogenic activities [5], and low intraspecific genetic variation. The intraspecific genetic variation in *A. nigrocincta* has been poorly studied. In recent years, molecular methods have been applied to study intraspecific genetic variation, species identification, and phylogenetic relationship among close-related taxa [6]. One technique used for this purpose is DNA barcoding. This method has been applied to conservation biology field because it provides information relevant to wildlife conservation management [7]. For animals, the area used for DNA barcoding is the cytochrome c oxidase I (COI) gene. In this study, we examined the intraspecific variation in *A. nigrocincta* to facilitate species identification and analyze their genetic distances.

## 2. Materials and Methods

### 2.1. Sample Collection

The adult worker bees were captured using a swingnet from an abandoned local garden in Mahakeret village, city of Manado, North Sulawesi province, Indonesia (1°28'53”N and 124°50'21”E). The location was situated at an altitude of about 40 meters above sea level. The average temperature at sample collection place (March 2019) was between 29°C and 32°C with humidity of 85–89%. The percentage of vegetation cover was 80%. The vegetation consisted mainly of bushes, grasses, and wild flowers such as *Sphagneticola trilobata* (L.) Pruski, *Tachytarpheta jamaicensis* (L.) Vahl., and *Mikania micrantha* Kunth.

### 2.2. Morphometric Analysis

The observed morphological characters included length of following parts: head (HD), antenna (AT), proboscis (PB), thorax (TO), abdomen (AB), fore-wing (FW), hind-wing (FL), midleg (ML), and hind-leg (HL) using a caliper [8].

### 2.3. Molecular Identification

#### 2.3.1. Sample Preparation, PCR, and Sequencing

Total DNA was extracted from coxa connected to the abdomen using ZR Tissue & Insect DNA MiniPrep™ (Zymo Research). The genomic DNA was cleaned with DNA Clean & Concentrator™-5 (DCC™-5) (Zymo Research) for the generation of high-quality DNA for PCR. The DNA barcode of COI region was amplified using Toyobo KOD FX Neo PCR Master Mix with primer pairs LCO1490 and HCO2198 [6]. The condition of PCR included 2 min of initial denaturation at 95°C followed by 35 cycles of denaturation at 98°C for 10 sec, annealing at 54°C for 30 sec, elongation at 68°C for 45 sec and additional extension for 5 min at 68°C. The PCR products were sequenced bidirectionally using the same PCR primer pairs at 1st BASE DNA Sequencing Services Malaysia.

#### 2.3.2. Analysis of COI Data

Chromatograms were subjected to the procedure as was done previously [9]. The clean COI sequence of the specimen was deposited in GenBank (http://www.ncbi.nlm.nih.gov). Identification was performed using BLAST identity search provided by the same platform. The clean sequences were aligned using Clustal O (1.2.1) multiple sequence alignment (http://www.ebi.ac.uk/Tools/msa/clustalo) with other allied honey bee COI sequences from different parts in Sulawesi retrieved from GenBank. The evolutionary history was inferred by the Maximum Likelihood (ML) method based on the Kimura 2-parameter (K2P) model [10]. Evolutionary analyses were conducted in MEGA v10.0.4 [11].

## 3. Results

### 3.1. Morphometric Analysis

**Figure 1:**
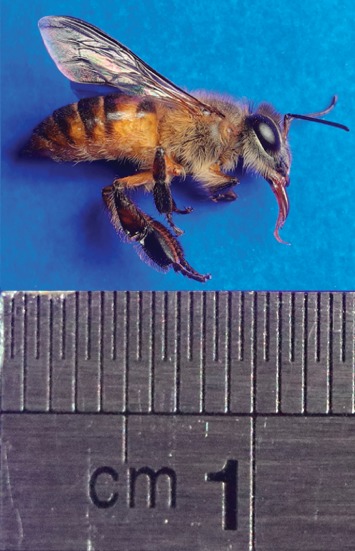
*Apis nigrocincta* CAL01captured in Mahakeret.

The photograph of honey bee specimen obtained from Manado Mahakeret is shown in Figure 1. The specimen hereinafter referred to as CAL01. The results of the nine major morphological characteristics of the honey bee depicted by acronyms as length of head (HD), antenna (AT), proboscis (PB), thorax (TO), abdomen (AB), fore-wing (FW), hind-wing (FL), midleg (ML), and hind-leg (HL) are presented in Table 1. These characters were compared with the characters of *A. nigrocincta* provided by Hadisoesilo et al. [12]. The morphometric analysis has been used previously to study genetic variability in honey bees [13].

### 3.2. Molecular Analysis

**Figure 2:**
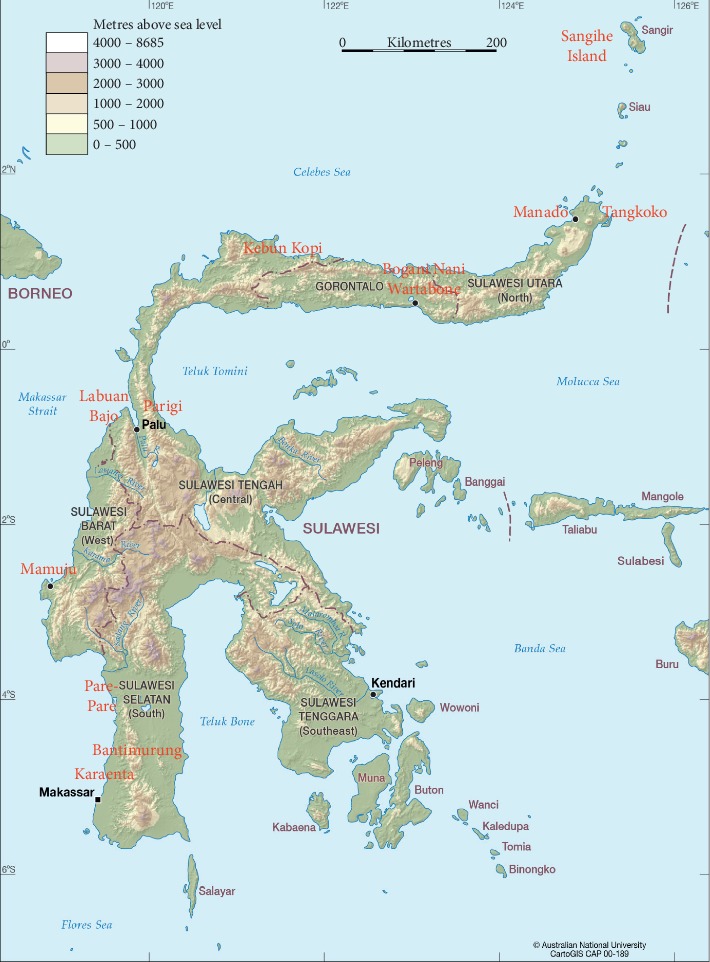
Map of Sulawesi Island showing the location of Manado city, Sangihe Island, and Labuan Bajo (Map source: Australian National University College of Asia and the Pacific).

The cytochrome oxidase I (COI) sequence of *A. nigrocincta* CAL01 has been deposited in GenBank with accession number MK880239. The complete BLAST search is presented in Table 2. The location of all Sulawesi specimens is shown in Figure 2. Because many sequences have 72% query cover, the sequences of some specimens were cut to match the length of other specimens. Even so, percent identity ranged between 92.24% and 100%. With a 72% query cover, the specimen CAL01 had a 100% identity with the DQ020233 specimen from Bogani Nani Wartabone National Park, Gorontalo, Indonesia. With a 100% cover query, the specimen CAL01 had a 92.24% identity with the KY834222 specimen from Islamabad, Pakistan.

**Figure 3:**
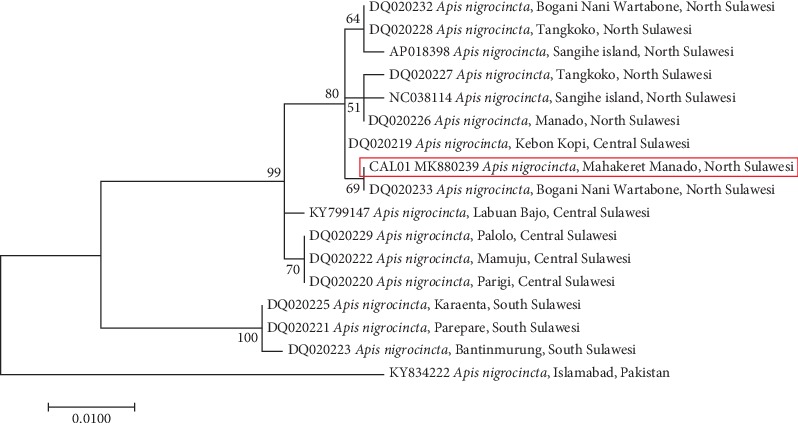
Molecular phylogenetic analysis by maximum likelihood method based on the Kimura 2-parameter model [5].

The molecular phylogenetic analysis by the ML method based on the K2P model is shown in Figure 3. The tree reveals that there were three main clades in Sulawesi *A. nigrocincta* specimens. Estimation of genetic distance amongst *A. nigrocincta* is shown in Table 3. Analysis was performed using the K2P model [10] integrated in MEGA v10.0.4 [11]. The intraspecific genetic distance ranged from 0.000 to 0.055 (excluding specimen from Pakistan).  Table 4 shows the polymorphic nucleotides of the COI. Twenty-six polymorphic sites were detected within *A. nigrocincta*. Variation of intraspecific COI gene showed that transition T ↔ C dominated the polymorphic pattern.

Estimation of substitution using the maximum composite likelihood method can be seen in Table 5. Different transition substitution rates are shown in bold and transversion substitution shown in italics. The sum of the values of *v* was equal to 100. The number of transition substitution was 86.89, while the number of substitution was 13.11. Therefore, the transition/transversion ratio (ti/tv) was 6.63. However, sampling size strongly influences the ti/tv. The estimated maximum likelihood ti/tv bias (*R*) was 9.05 under the K2P model [10].

## 4. Discussion

Referring to the morphometric analysis conducted by Hadisoesilo et al. [12], as well as other physical characteristics, it is believed that the honey bee being studied was *Apis nigrocincta* (Figure 1). Nevertheless, this method still has short comings due to lack of expertise from the researchers. Thus, a simpler but more accurate method was needed. One method used to identify insects that has been widely used is DNA barcoding using the COI gene. This COI gene has been widely used in evaluating inter- and intraspecific diversity in insects [14]. It is also used to complement traditional morphological-based identification to get more accurate species identification results [6]. Several studies of DNA barcoding in honey bees using the COI gene have been carried out by several researchers [5, 15–18]. Therefore, molecular-based identification was still carried out in this research. After being confirmed using the COI gene, it was ascertained that the specimen being studied was *A. nigrocincta*.

As shown in Figure 3, the specimens were grouped based on location. The first group was from North Sulawesi, the second was from Central Sulawesi, and the third was from South Sulawesi. However, the specimen DQ020219 from Kebun Kopi Central Sulawesi was clustered together with the North Sulawesi group. This can be because the location of Kebun Kopi is very close to Bogani Nani Wartabone National Park which is situated in North Sulawesi. Low nucleotide variations in the data presented in this research in Sulawesi specimens indicated that there was no geographical isolation, hence the gene flew among unrestricted populations, especially in nearby locations.

The specimen KY834222 from Islamabad Pakistan was observed to be out of the Sulawesi group. In the phylogenetic tree created by involving its sister species, *A. cerana*, it was seen that specimen KY834222 was grouped with *A. cerana japonica* (data not shown). This phenomenon was supported by studies conducted by Damus and Otis [19] which stated that several subspecies of *A. cerana* and *A. cerana japonica* were confirmed as being distinct from the rest of *A. cerana*. This indicates that *Apis nigrocincta* found in Pakistan was most likely the part of the group *A. cerana japonica*. Another finding showed that *A. nigrocincta* from Sulawesi mainland and Sangihe Island were embedded in the *A. cerana* group [20]. The phylogenetic analysis was consistent with morphological and molecular evidence, indicating that *A. nigrocincta* had similarities with *A. cerana* [21, 22].

The closest genetic distance was 0.000, between haplotype CAL01 and DQ020233 from Bogani Nani Wartabone National Park, Gorontalo (Indonesia). The farthest genetic distance was 0.082, between haplotype CAL01 and KY834222 from Islamabad (Pakistan), and 0.052 with DQ020223 from Bantimurung, South Sulawesi (Indonesia). The genetic distance was 0.055 between NC038114 from Sangihe Island North Sulawesi (Indonesia) and DQ020223. With this value, Takahasi et al. [23] stated that *A. nigrocincta* maintained a high specific genetic diversity on Sulawesi Island.

The result of genetic distance calculation indicated that although the tested specimens were derived from remote locations, it was still within the range of intraspecific variation, except with specimen from Pakistan. Maximum pairwise distance of *A. mellifera* in 10 locations across China and Pakistan was 0.039 [18]. The result amongst Pakistan's haplotypes was 0.027 and China's haplotypes was 0.010. The maximum pairwise distance 0.053 among *A. cerana* was detected between Flores' and Taiwan's haplotypes [24]. The closer the genetic distance, the closer the kinship among the organisms being compared [25]. Different geographical conditions and remote locations can cause fairly high genetic variation in a species and are usually characterized by morphological differences [26, 27]. The lack of data collection on various species and the dependence of the morphological identification process have led to debate for species that have similar morphological structures but are classified as different species due to genetic variation [28, 29]. The greater the value of genetic distance between populations or individuals, the more isolated they are from one another. Genetic distance indicates the possibility of the influence of geographical isolation on a population [30, 31].

No information was provided earlier on the polymorphic sites of *A. nigrocincta*. However, six polymorphic sites were detected in *Bombus koreanus* in China [32] and *Apis mellifera* in United States [33]. The transition T ↔ C was found dominating the variation in *A. cerana* in India [34]. Due to molecular mechanism, transitions are more often found in a higher frequency than transversions. Furthermore, the transition tends to produce less amino acid substitution. Therefore, the transition tends to be more stable, and is a silent substitution as a single nucleotide polymorphism in one population [25].

The ability of COI gene to differentiate between *A. nigcrocinta* group from different locations in Sulawesi has been demonstrated in this study. This molecular marker was also able to identify *A. nigrocincta* successfully based on the similarity sequence with other *A. nigrocincta* specimens. This finding provides valuable information on the effective use of COI as molecular marker in research on phylogenetic studies.

## 5. Conclusions

The current study assessed the intraspecific genetic variation in *A. nigrocincta* using COI gene sequences. The results showed that there were three main clades of Sulawesi specimen, namely, North, Central, and South Sulawesi. This finding suggested that genetic variation in *A. nigrocincta* in Sulawesi is still in the category of intraspecific variation.

## Figures and Tables

**Table 1:** Morphometric analysis of *Apis nigrocincta* CAL01.

Characters	Length (mm)	Length (mm) [12]
Head (HD)	3.57 ± 0.08	N/A
Antenna (AT)	3.06 ± 0.08	N/A
Proboscis (PB)	4.15 ± 0.18	4.98
Thorax (TO)	3.03 ± 0.04	N/A
Abdomen (AB)	6.04 ± 0.04	N/A
Fore-wing (FW)	8.03 ± 0.10	8.12
Hind-wing (FL)	3.30 ± 0.40	2.73
Midleg (ML)	3.50 ± 0.09	N/A
Hind-leg (HL)	8.04 ± 0.14	7.31

**Table 2:** Complete BLAST search.

Max. score	Total score	Query cover (%)	E value	Percent identity (%)	Accession	Location
1099	1099	100	0.0	99.50	AP018398.1	Sangihe Island, North Sulawesi, Indonesia
1099	1099	100	0.0	99.50	NC_038114.1	Sangihe Island, North Sulawesi, Indonesia
1083	1083	100	0.0	99.01	KY799147.1	Labuan Bajo, Central Sulawesi, Indonesia
856	856	100	0.0	92.24	KY834222.1	Islamabad, Pakistan
804	804	72	0.0	100.00	DQ020233.1	Bogani Nani Wartabone N.P, Gorontalo, Indonesia
798	798	72	0.0	99.77	DQ020219.1	Kebon Kopi, Central Sulawesi, Indonesia
793	793	72	0.0	99.54	DQ020232.1	Bogani Nani Wartabone N.P., Gorontalo, Indonesia
793	793	72	0.0	99.54	DQ020228.1	Tangkoko Batuangus N.P., North Sulawesi, Indonesia
793	793	72	0.0	99.54	DQ020226.1	Manado, North Sulawesi, Indonesia
787	787	72	0.0	99.31	DQ020227.1	Tangkoko Batuangus N.P., North Sulawesi, Indonesia
776	776	72	0.0	98.85	DQ020229.1	Palolo, Central Sulawesi, Indonesia
776	776	72	0.0	98.85	DQ020222.1	Mamuju, South Sulawesi, Indonesia
776	776	72	0.0	98.85	DQ020220.1	Parigi, Central Sulawesi, Indonesia
688	688	72	0.0	95.17	DQ020225.1	Karaenta, South Sulawesi, Indonesia
688	688	72	0.0	95.17	DQ020221.1	Parepare, South Sulawesi, Indonesia
682	682	72	0.0	94.94	DQ020223.1	Bantinmurung, South Sulawesi, Indonesia

**Table 3:** Estimation of evolutionary divergence between selected haplotypes.

	Haplotypes	1	2	3	4	5	6	7	8	9	19	11	12	13	14	15	16	17
1	AP018398																	
2	NC038114	0.009																
3	KY799147	0.014	0.014															
4	KY834222	0.085	0.085	0.080														
5	**CAL01**	**0.007**	**0.007**	**0.011**	**0.082**													
6	DQ020233	0.007	0.007	0.011	0.082	**0.000**												
7	DQ020219	0.005	0.005	0.009	0.080	**0.002**	0.002											
8	DQ020232	0.002	0.007	0.012	0.083	**0.005**	0.005	0.002										
9	DQ020228	0.002	0.007	0.012	0.083	**0.005**	0.005	0.002	0.000									
10	DQ020226	0.007	0.002	0.012	0.083	**0.005**	0.005	0.002	0.005	0.005								
11	DQ020227	0.009	0.005	0.009	0.080	**0.007**	0.007	0.005	0.007	0.007	0.002							
12	DQ020229	0.014	0.014	0.005	0.078	**0.012**	0.012	0.009	0.012	0.012	0.012	0.009						
13	DQ020222	0.014	0.014	0.005	0.078	**0.012**	0.012	0.009	0.012	0.012	0.012	0.009	0.000					
14	DQ020220	0.014	0.014	0.005	0.078	**0.012**	0.012	0.009	0.012	0.012	0.012	0.009	0.000	0.000				
15	DQ020225	0.048	0.052	0.043	0.075	**0.050**	0.050	0.048	0.045	0.045	0.050	0.048	0.043	0.043	0.043			
16	DQ020221	0.048	0.052	0.043	0.075	**0.050**	0.050	0.048	0.045	0.045	0.050	0.048	0.043	0.043	0.043	0.000		
17	DQ020223	0.050	0.055	0.045	0.078	**0.052**	0.052	0.050	0.048	0.048	0.053	0.050	0.045	0.045	0.045	0.002	0.002	

**Table 4:** Haplotypes and polymorphic nucleotides of the COI.

The COI polymorphic nucleotide sites																											
Haplotypes/Gen Bank acc. numbers	Location	1873	1954	1961	1963	1984	1988	2017	2032	2053	2059	2065	2086	2096	2098	2113	2143	2015	2018	2027	2028	2036	2042	2052	2076	2078	2090
AP018398	Sangihe, North Sulawesi	**C**	C	C	A	A	C	A	A	C	T	T	A	C	T	C	G	T	T	**T**	C	T	T	T	C	T	T
DQ020232	Bogani Nani WB, North Sulawesi	T	C	C	A	A	C	A	A	C	T	T	A	C	T	C	G	T	T	**T**	C	T	T	T	C	T	T
DQ020228	Tangkoko, North Sulawesi	T	C	C	A	A	C	A	A	C	T	T	A	C	T	C	G	T	T	**T**	C	T	T	T	C	T	T
NC038114	Sangihe, North Sulawesi	T	C	C	A	A	C	A	A	C	T	T	A	C	T	C	G	T	**C**	C	C	**C**	T	T	C	T	T
DQ020226	Manado, North Sulawesi	T	C	C	A	A	C	A	A	C	T	T	A	C	T	C	G	T	T	C	C	**C**	T	T	C	T	T
DQ020227	Tangkoko, North Sulawesi	T	C	C	A	A	T	A	A	C	T	T	A	C	T	C	G	T	T	C	C	**C**	T	T	C	T	T
CAL01	Manado, North Sulawesi	T	C	C	A	A	**C**	A	A	C	T	T	A	C	T	C	G	T	T	C	C	T	T	T	C	T	T
DQ020233	Bogani Nani WB, North Sulawesi	T	C	C	A	A	**C**	A	A	C	**C**	T	A	C	T	C	G	T	T	C	C	T	T	T	C	T	T
DQ020219	Kebun Kopi, Central Sulawesi	T	C	C	A	A	**C**	A	A	C	**C**	T	A	C	T	C	G	T	T	C	C	T	T	T	C	T	T
KY799147	Labuan Bajo, Central Sulawesi	T	C	C	A	A	T	A	A	C	T	T	A	C	T	C	**A**	T	T	C	C	T	T	T	C	T	**C**
DQ020229	Palolo, Central Sulawesi	T	C	C	**G**	A	T	A	A	C	T	T	**T**	C	T	C	**A**	T	T	C	C	T	T	T	C	T	**C**
DQ020222	Mamuju, South Sulawesi	T	C	C	**G**	A	T	A	A	C	T	T	A	C	T	C	**A**	T	T	C	C	T	T	T	C	T	**C**
DQ020220	Parigi, Central Sulawesi	T	C	C	**G**	A	T	A	A	C	T	T	A	C	T	C	**A**	T	T	C	C	T	T	T	C	T	**C**
DQ020225	Karaenta, South Sulawesi	T	**T**	**T**	A	**G**	T	**T**	**T**	**T**	T	**A**	A	**T**	**A**	**T**	**A**	T	T	**T**	**T**	T	**A**	**C**	**T**	**A**	**C**
DQ020221	Parepare, South Sulawesi	T	**T**	**T**	A	**G**	T	**T**	**T**	**T**	T	**A**	A	**T**	**A**	**T**	**A**	T	T	**T**	**T**	T	**A**	**C**	**T**	**A**	**C**
DQ020223	Bantinmurung, South Sulawesi	T	**T**	**T**	A	**G**	T	**T**	**T**	**T**	T	**A**	A	**T**	**A**	**T**	**A**	**C**	T	**T**	**T**	T	**A**	**C**	**T**	**A**	**C**

Transversion (Tv)/transition (Ts)		Ts	Ts	Ts	Ts	Ts	Ts	Tv v	Tv	Tv	Ts	Tv	Tv	Ts	Tv	Ts	Ts	Ts	Ts	Ts	Ts	Ts	Tv	Ts	Ts	Tv	Ts

**Table 5:** Estimation of the nucleotide substitution pattern using the maximum composite likelihood method.

	A	T	C	G
A	—	2.68	1.05	**8.34**
T	2.16	—	**14.46**	0.66
C	2.16	**36.82**	—	0.66
G	**27.27**	2.68	1.05	—

## Data Availability

The data related to this article are available from the corresponding author upon request.
